# Brain–Computer Interface Spellers for Communication: Why We Need to Address Their Security and Authenticity

**DOI:** 10.3390/brainsci10030139

**Published:** 2020-03-02

**Authors:** Anirban Dutta

**Affiliations:** Department of Biomedical Engineering, University at Buffalo, Buffalo, NY 14260-1660, USA; anirband@buffalo.edu; Tel.: +1-716-645-9161

Brain–Computer Interfaces (BCI) have witnessed significant research and development in the last 20 years where the main aim was to improve their accuracy and increase their information transfer rates (ITRs), while still making them portable and easy to use by a broad range of users. A recent review article on BCI-spellers by Rezeika and colleagues [1] in the Brain Sciences journal provided an overview of different BCI paradigms, including P300, steady-state visual evoked potential (SSVEP), and motor imagery (MI), which are currently being used as communication channels for spelling. In these BCI-spellers, the output is provided as a graphical representation of letters, numbers, and symbols, or an audio output. The front-end of the BCI speller is the Graphical User Interface (GUI) which is very important according to Rezeika and colleagues [1]. Indeed, the human-in-loop driving the BCI-speller receives feedback using the GUI (except in the case of motor imagery BCI), which therefore needs to be customized according to the residual function of the end-user. For example, SSVEP-based BCI will require a functional retina where the fidelity of the event-related potential (ERP) can be supplemented by auditory stimulation in a multimodal ERP-based BCI. However, the challenge is in the multi-modal sensor fusion, e.g., in optimizing the visual-to-auditory delay in multimodal spellers that combine visual and auditory stimulation [2]. Such hybrid BCI systems may not only address subject-specific residual function but can also provide a more accurate and faster communication channel by combining different ERPs [1]. An electroencephalogram (EEG)-based BCI speller has recently achieved ITRs of up to 5.32 bits per second and spelling rates up to 60 characters per minute [3]. Also, soft electrode systems for EEG have been developed for continuous long-term use [4]. With such advancement of the hardware and software for fast EEG-based communication channel for spelling, practical and naturalistic applications of BCI-spellers in daily living have become a reality, e.g., as a communication channel to send messages to the external world. In such practical applications, an important requirement for the BCI-speller will be to provide secure and authentic communication. In fact, user authentication (e.g., in the GUI) and resistance to attacks (e.g., in the BCI system) will have to be in-built for use as an official communication channel in real life.

EEG-based BCI-spellers are currently most popular [3] due to the portability, non-invasiveness, and low-cost of consumer EEG systems [5]. Let us take the example of BCI-speller that uses SSVEPs that are generated by voluntary user attention to flickering stimulus. Here, each character will have its own unique frequency (usually between 3.5 Hz and 75 Hz), and the evoked EEG will have the same frequency as the attended stimulus (and its harmonics). Classification of the SSVEPs with a narrow frequency range required in high-speed spellers [3] leverages advanced machine learning techniques to identify the user-intended character which can provide a fast asynchronous BCI [3]. However, machine learning techniques can be susceptible to adversarial attacks which have been demonstrated for convolution neural network (CNN) [6,7], and regression model [8] classifiers. In a white-box attack [8], all the information about the machine learning algorithm is known such that small perturbations can be designed based on direction sensitivity estimation [9] as an input noise to change the classifier output. In a black-box attack, the attacker knows nothing about the machine learning system [6,7] but may have some access to the training dataset or features. For example, in Figure 1, in the case of SSVEP-based BCI-speller, if the attacker knows the frequency modulation (i.e., features) of their intended characters then electromagnetic (EM) noise with those characteristic perturbations can be delivered to the environment of the end user which can be transferrable [10]. This attacker-delivered EM noise (as adversarial template) can be picked by the EEG electrodes driving the BCI-speller to output the attacker-intended characters (e.g., based on template-based decoding [3]) especially with low signal-to-noise ratio (SNR) consumer EEG systems [5]. Therefore, the design of the BCI-speller needs to not only address countermeasures to the adversarial perturbations and its transferability [11], but the EEG hardware design also needs to account for improved SNR and adequate shielding to reject adversarial EM noise attacks. 

Design of the BCI-speller can leverage unique subject-specific signatures to improve its security and authenticity, e.g., inter-subject variability resulting from the complex EEG activity generating SSVEPs can provide subject-specific features [12] to prevent transferability [11]. Such subject-specific feature-set can be identified during the calibration phase [13], e.g., a recent high-speed BCI-speller leveraged subject-specific stable latency (reflected in the phase) of single-trial SSVEP and used joint frequency-phase modulation method to enhance the discriminability [3]. Such joint frequency-phase modulation method can be more robust to adversarial feature perturbations where other unique subject-specific features can be added as a unique signature to make asynchronous BCI-spellers robust to black-box attacks. In conclusion, future research and development of the hardware and the software for the EEG-based BCI-speller, and BCIs in general, need to address its security and authenticity as a robust communication channel for real-life applications. 

## Figures and Tables

**Figure 1:**
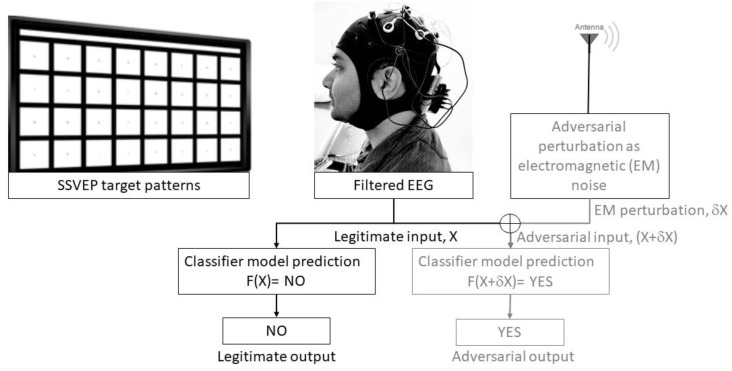
Adversarial crafting for SSVEP-based BCI-speller: electromagnetic (EM) noise of adversarial perturbations with the known frequency modulation (SSVEP feature) can be delivered to the environment of the end user which can lead to misclassification of the legitimate input (for output NO) to the adversarial output (=YES) by the classifier model (F).
